# Factors Affecting the Outcomes of Patients with Malignant Rhabdoid Tumors: A Population-Based Study

**DOI:** 10.7150/ijms.51186

**Published:** 2021-01-01

**Authors:** Wen Cai, Xue Liu, Weiting Ge, Dehao Wu, Junxi Xu, Rui Bai, Hanguang Hu

**Affiliations:** 1 Departments of Gastroenterology, The Second Affiliated Hospital, Zhejiang University School of Medicine, Hangzhou, Zhejiang 310009, P.R. China; 2 Departments of Medical Oncology, The Second Affiliated Hospital, Zhejiang University School of Medicine, Hangzhou, Zhejiang 310009, P.R. China; 3 Cancer Institute (Key Laboratory of Cancer Prevention and Intervention, China National Ministry of Education), The Second Affiliated Hospital, Zhejiang University School of Medicine, Hangzhou, Zhejiang 310009, P.R. China

**Keywords:** Malignant rhabdoid tumor, SEER, Risk factors, Epidemiology

## Abstract

**Objective:** Malignant rhabdoid tumor (MRT) is a rare but aggressive malignancy. It has been a long time since data on this tumor have been updated.

**Methods:** We retrospectively reviewed patients from the SEER database who were pathologically diagnosed with MRT and analyzed incidence rates, clinical features and survival using Stata 12.0.

**Results:** In total, 544 patients were included in the epidemiological analysis. There were two peak periods of MRT incidence: patients younger than 4 years and those older than 70 years. Further survival analysis showed that the survival of children (especially younger than 1 year) was markedly worse than that of adults (*P*<0.01), and different primary sites were associated with different age groups and survival outcomes. The central nervous system (CNS) was the most common primary site (50.00%), followed by the kidney (15.66%). Patients with MRTs that originated from the digestive system experienced worse survival outcomes than those with MRTs originating from other locations. Primary site surgery conferred survival benefits to patients with renal and digestive system MRTs (HR = 0.06, CI: 0.02-0.23, *P*<0.01; HR=0.10, CI: 0.02-0.48, *P*<0.01), whereas radiotherapy conferred benefits to patients with CNS, bone and soft tissue MRTs (HR=0.22, CI: 0.15-0.34, *P*<0.01; HR=0.44, CI: 0.21-0.90 *P*=0.03).

**Conclusions:** Our results indicate that age and the primary site of MRT are critical clinical factors that affect patient survival and treatment choices. Primary site tumor resection should be considered for renal and digestive system MRTs, and systematic therapy, including surgery and radiotherapy, should be recommended for the treatment of CNS, bone and soft tissue MRTs.

## Introduction

Malignant rhabdoid tumors (MRTs) were initially described by Haas JE in 1981 in patients in their early childhood with primary renal tumors 1, 2. Subsequent studies have reported the same type of tumors in extrarenal regions, such as the central nervous system (CNS) and miscellaneous soft tissue locations 3, 4. Several hospital-based series have established that the prevalence of CNS MRTs is 1% to 2% among pediatric patients with brain tumors 5. A study from Australia reported an age-standardized incidence rate of 1.38 per 1,000,000 person-years in children 6. With the recent realization and diagnosis of these diseases, especially MRTs located in primary CNS sites, the incidence and prevalence rates of CNS MRTs are increasing. However, the incidence rate of MRTs among different age groups or other primary sites remains unclear. The data summarized in our study describe the clinical features, survival outcomes and treatment options for patients with MRTs from all primary sites. Furthermore, the incidence rate of MRTs among different age groups was calculated to present clinicians with an overview of MRTs to provide guidance when faced with these patients.

In the year 2000, CNS MRTs were introduced to the World Health Organization (WHO) brain tumor classification and the International Classification of Diseases for Oncology, third edition (ICD-O-3) 7, which reduced the rate of misdiagnosis of this aggressive tumor and distinguished MRTs from primitive neuroectodermal tumors (PNETs) and synovial sarcoma (SS). With the development of gene sequencing technology, an increasing number of studies have confirmed that the tumor suppressor gene INI1/SMARCB1, which is ubiquitously expressed in normal tissue, is completely lost in MRTs and atypical teratoid/rhabdoid tumors (ATRTs). Recently, patients suspected of having MRTs have been encouraged to undergo tests of relevant gene function. Additionally, there is no consensus on the standard treatment for MRTs; thus, our respective study provides additional ideas and experience for designing clinical trials. Because of the poor prognosis of MRTs, physicians treat these patients using multimodality therapy, with radiotherapy being considered an essential component; however, whether radiotherapy is effective for MRTs at every primary site and the effectiveness of radiotherapy combined with surgery remain unknown 3.

Therefore, in this study, we selected patients from the Surveillance, Epidemiology, and End Results (SEER) database, an authoritative cancer information database that was initiated in 1974 that comprises information on cancer survival 8, and excluded patients diagnosed before the year 2000 to reduce the misdiagnosis rate so that we could further analyze and describe the clinical risk factors and therapy choices available for patients with MRTs.

## Results

### Incidence rate and patient characteristics

We identified 544 patients from the SEER 18 database who were diagnosed with MRTs between 2000 and 2014 and analyzed the incidence rate. We calculated the 14-year incidence rate of each age group, and the results showed that patients younger than 4 years had an obviously higher incidence rate than older patients (1.83 per 100,000 people, based on the standard population from the SEER database), and patients older than 70 years accounted for the other peak in incidence, as shown in Fig 2 (0.2 per 100,000 people, based on the standard population from the SEER database).

To further analyze the epidemiological and clinicopathological characteristics of these patients, we evaluated their data, excluding 38 patients whose data did not meet our qualifications. Basic patient information is summarized in Table 1, grouped according to the different primary sites of MRTs. The median overall survival (mOS) time was only months (less than one year) for tumors at each of the primary sites, which suggests that MRTs are highly aggressive, regardless of the site. The CNS was the most common primary site (50.00%), followed by the kidney (15.66%). We separated digestive system MRTs from MRTs located in other primary sites, as MRTs located in the digestive system accounted for 26.67% of all MRTs in the remaining primary sites and were associated with worse survival than MRTs located in other primary sites (mOS: 5.58 months). The tumors located at other uncommon sites were too few to perform further analyses. Regarding treatment, patients who underwent surgery or radiotherapy for the primary tumor had varying primary sites. There was no significant difference in terms of sex or race among the patients with MRTs located at different primary sites. There was a significant difference in marital status among patients, which was expected according to the different age groups studied.

### Risk factors for MRTs located at different primary sites

Based on the initial analysis of the epidemiological and clinicopathological characteristics of the patients, we further explored the risk factors for MRTs at different primary sites using both multivariate and univariate analyses. The results are presented in Tables 2 and 3.

In patients with renal MRTs, the univariate analysis suggested that age and primary site surgery affected patient survival. Furthermore, the multivariate analysis showed that when we adjusted for sex, race, marital status and SEER stage, primary site surgery decreased the risk of death (hazard ratio, HR=0.06, confidence interval, CI: 0.02-0.17, *P*<0.01), while radiation therapy showed a tendency to increase the risk of death (HR=2.34, CI: 1.01-5.43, *P*=0.05). Furthermore, patients ranging in age from 1 to 18 years had better survival than patients who were younger than 1 year (HR=0.23, CI: 0.09-0.58,* P*<0.01).

In patients with CNS MRTs, the univariate analysis suggested that age and radiation therapy affected patient outcomes. Furthermore, the multivariate analysis showed that when we adjusted for age, sex, race, SEER stage and marital status, radiation therapy was the only factor that decreased the risk of death (HR=0.22, CI: 0.15-0.34,* P*<0.01).

Radiotherapy also conferred benefits to patients with bone and soft tissue MRTs when we adjusted for sex, race, marital status and SEER stage (HR=0.44, CI: 0.21-0.90 *P*=0. 03). Patients ranging in age from 1 to 18 years had better survival than patients who were younger than 1 year (HR=0.34, CI: 0.14-0.84, *P*=0.02).

In patients with digestive system MRTs, the univariate analysis suggested that primary site surgery affected patient outcomes (HR=0.10, CI: 0.02-0.48, *P*<0.01). In the multivariate analysis, when we adjusted for age, sex, race, SEER stage and marital status, primary site surgery was the only factor that decreased the risk of death (HR=0.03, CI: 0.01-0.28, *P*<0.01).

### Survival analysis

A total of 335 patients had complete overall survival data, but 54 patients died within a month of diagnosis. For these patients, the survival time in months was zero; thus, they could not be included in the Kaplan-Meier curve. According to the risk factors we obtained from the previous analysis, further survival analysis was performed as follows.

### Survival outcomes across different age groups

We observed that most patients, except those with tumors originating from the CNS, were adults (older than 18 years), but patients younger than 1 year showed the highest incidence rate. Therefore, we further divided the patients into three age groups (younger than 1 year, 1 to 18 years and older than 18 years) to perform further survival analysis using the Kaplan-Meier method and log-rank test, the results of which are presented in Fig 3A. This figure shows that patients younger than 1 year had obviously worse survival than older patients, and adult patients had better survival than younger patients. Further exploration of the relationship between age groups and primary sites is presented in Fig 3B. The CNS was the most common site of MRTs in patients younger than 18 years, while there were several common sites among adults.

### Survival outcomes and treatment choices for MRTs at different primary sites

We summarize the different treatment options available to patients with MRTs located at different primary sites in Fig 4. We could not include chemotherapy data because the SEER database provides no specific data on the use of chemotherapy. However, data on whether primary site surgery and radiotherapy were performed were available. Therefore, four groups of patients who received different treatments were generated: both surgery and radiotherapy, surgery alone, radiotherapy alone, and no surgery or radiotherapy. In total, 80.43% of patients received primary surgery, 62.45% received radiotherapy, and 32.61% received both surgery and radiotherapy. Further survival analysis suggested that patients who received both surgery and radiotherapy had better survival than those who received other types of treatments (Fig 5A). Then, we performed survival analysis for MRTs located at each primary site; the results are presented in Fig 5B-F.

Considering the risk factors for this disease, we suggest that primary site surgery is vital and improves the overall survival of patients with renal and digestive system MRTs, while systemic therapy, including surgery and radiotherapy, may improve the survival of patients with CNS, bone and soft tissue MRTs.

## Discussion

Our study summarizes data on 544 patients diagnosed with MRTs between 2000 and 2014 from the SEER database. We present an overview of all types of MRTs, which makes our study distinguishable from others that contain data only on MRTs located at common sites, such as the CNS or kidneys. The results showed that the CNS was the most common primary site, followed by the kidney, consistent with previous studies 6, 9. Recent research that has focused on MRTs is limited, except for research on MRTs located at common primary sites. In our study, we investigated digestive system MRTs, which comprise 26.67% of the MRTs located in uncommon primary sites, and found that MRTs originating from uncommon sites such as the digestive system were more aggressive than MRTs located at common primary sites; the mOS time of these patients was only 5.58 months. However, we did not identify any useful factors that could predict the prognosis of patients with MRTs located at uncommon primary sites because of the limited number of such patients.

Previous studies have reported that age could be a critical factor reflecting patient outcomes. A study of 31 children with CNS MRTs from St. Jude Children's Research Hospital concluded that over a period of 19 years, children younger than 3 years had significantly poorer survival than older children 10. Tomlinson et al. analyzed children with renal MRTs from the National Wilms Tumor Studies and found that children younger than 2 years at diagnosis had a significantly poorer outcome than older children 11. In our study, we separated patients into three groups according to the incidence rate and median age at diagnosis. The results suggested that adults experienced better survival, and patients younger than 1 year experienced markedly worse survival, which is concordant with the results from previous studies 3, 11. Therefore, we further investigated the primary sites of MRTs according to age group and calculated the risk of death across different age groups to provide valuable insight and guidance for future studies.

We also calculated the incidence rate of MRTs among these patients, and there were two obvious peaks. Due to limitations in SEER*Stat, we could not calculate the prevalence using smaller age ranges because we could not analyze a standard population. One peak occurred among patients younger than four years of age, consistent with previous reports, which has received much attention. The other peak appeared among patients older than 70 years, which is the first time this finding has been reported. Furthermore, we found that patients older than 70 years at the time of MRT diagnosis were likely to have MRTs located at uncommon primary sites, such as the digestive system. The analysis of age focused on the incidence rate and distribution of primary sites, making our study unique compared to previous studies. We call for close attention to be paid to MRTs originating from these uncommon sites and for further comparisons of the differences between children and adults with MRTs. The diagnosis of MRTs originating from uncommon primary sites may be challenging due to the lack of experience of clinicians in diagnosing these tumors. The INI1 mutation is a hallmark of MRTs and is critical in distinguishing MRTs from other rare tumor types. Immunohistochemistry (IHC) staining for INI1 is currently the gold standard for MRT diagnosis. INI1 positivity in a pathological report warrants close attention, as it indicates MRTs in uncommon primary sites. Unfortunately, studies in which clinical and pathological information is combined to analyze risk factors are lacking due to the limited number of patients, and most databases, such as the SEER database, have no records of IHC staining or genetic information. Recently, the identification of *h*SNF5 gene mutation has received much attention. Many basic science studies have focused on this mutation as a new therapeutic target 12-14.

The effectiveness and safety of surgery and radiotherapy for MRT remain controversial 15-18. There are two main reasons: first, previous studies combined all MRT patients, and second, the treatment options vary due to the lack of standardized guidelines, which leads to an inability to compare the effectiveness of different treatments. In our study, we separated patients into groups based on MRTs located at different primary sites and performed both univariate and multivariate analyses to present credible results. The conclusions drawn from our study are different from those drawn from previous studies 10, 19, which have encouraged radiotherapy for MRTs regardless of the primary site; in our study, radiotherapy conferred survival benefits to patients with CNS, bone and soft tissue MRTs. Nevertheless, no standardized methods or doses of radiotherapy for MRTs have been proposed because most of the analyses have been performed on data from the SEER database; thus, there is no way to determine whether the radiation administered was palliative or therapeutic or why treatments were or were not administered. Moreover, many studies have reported that patients with CNS MRTs could gain survival benefits from surgery, but this difference was not significant in our multivariate analysis. One limitation is the use of data from the SEER database, as stated above. Another limitation is that studies involving CNS MRTs usually focus only on the brain, while the group of patients with CNS MRTs in our study exhibited both brain and spinal cord MRTs.

On the other hand, surgery is a vital anticancer strategy. Most patients with brain MRT undergo primary tumor resection and gain survival benefits. Whether primary site surgery for MRTs located at other primary sites congers survival benefits has rarely been reported. In our study, patients with CNS MRTs gained more survival benefits by undergoing both primary surgery and radiotherapy than by undergoing primary site surgery alone. Furthermore, patients with MRTs originating from the kidneys or digestive system are recommended to undergo surgery. Taking all the above into consideration, we conclude that primary site surgery is vital and improves overall survival among patients with renal and digestive system MRTs, while systemic therapy, including surgery and radiotherapy, may improve patient survival for those with CNS, bone and soft tissue MRTs at specific sites.

Our study was a retrospective database study that focused on clinical factors affecting the overall survival of patients with MRTs and has unavoidable limitations; thus, all of these conclusions should be investigated further in prospective studies. The two main limitations are related to the shortcomings of the SEER database, as we could not obtain IHC, genetic or chemotherapy data from this database. Almost all MRTs show a complete loss of SMARCB1/INI1 expression 20, and according to a recent report, survival outcomes have been shown to be significantly poorer in those with SMARCA4 mutation than in those with the more common mutation in SMARCB1 21. Therefore, we call for more data, such as essential IHC or genetic data, to be recorded in databases, and studies that can combine clinical and pathological factors to construct risk models for patients will be meaningful. Chemotherapy is also a factor that may have a significant effect on survival. However, the reality is that the effect of chemotherapy is difficult to calculate or adjust for even if we obtain the appropriate data 22, as very different regimens are attempted for these uncommon diseases. The most reasonable way to describe the choice of chemotherapy is to review the literature, such as previous studies with large sample sizes, or to design prospective studies, such as clinical trials.

## Conclusion

Based on the results of the above clinical factor analyses, we observed that two age groups shared a high incidence rate of MRTs: patients younger than 4 years and those older than 70 years. Younger patients had worse survival than older patients. Different treatment choices should be considered for MRTs located at different primary sites. Primary site tumor resection should be considered for renal and digestive system MRTs, and systemic therapy, including surgery and radiotherapy, should be recommended for the treatment of CNS, bone and soft tissue MRTs. More specific information, such as IHC or genetic data, doses of radiotherapy and chemotherapy and the sequence of radiotherapy, surgery and chemotherapy, must be recorded in national databases.

## Methods

### Data source

**Figure 1:**
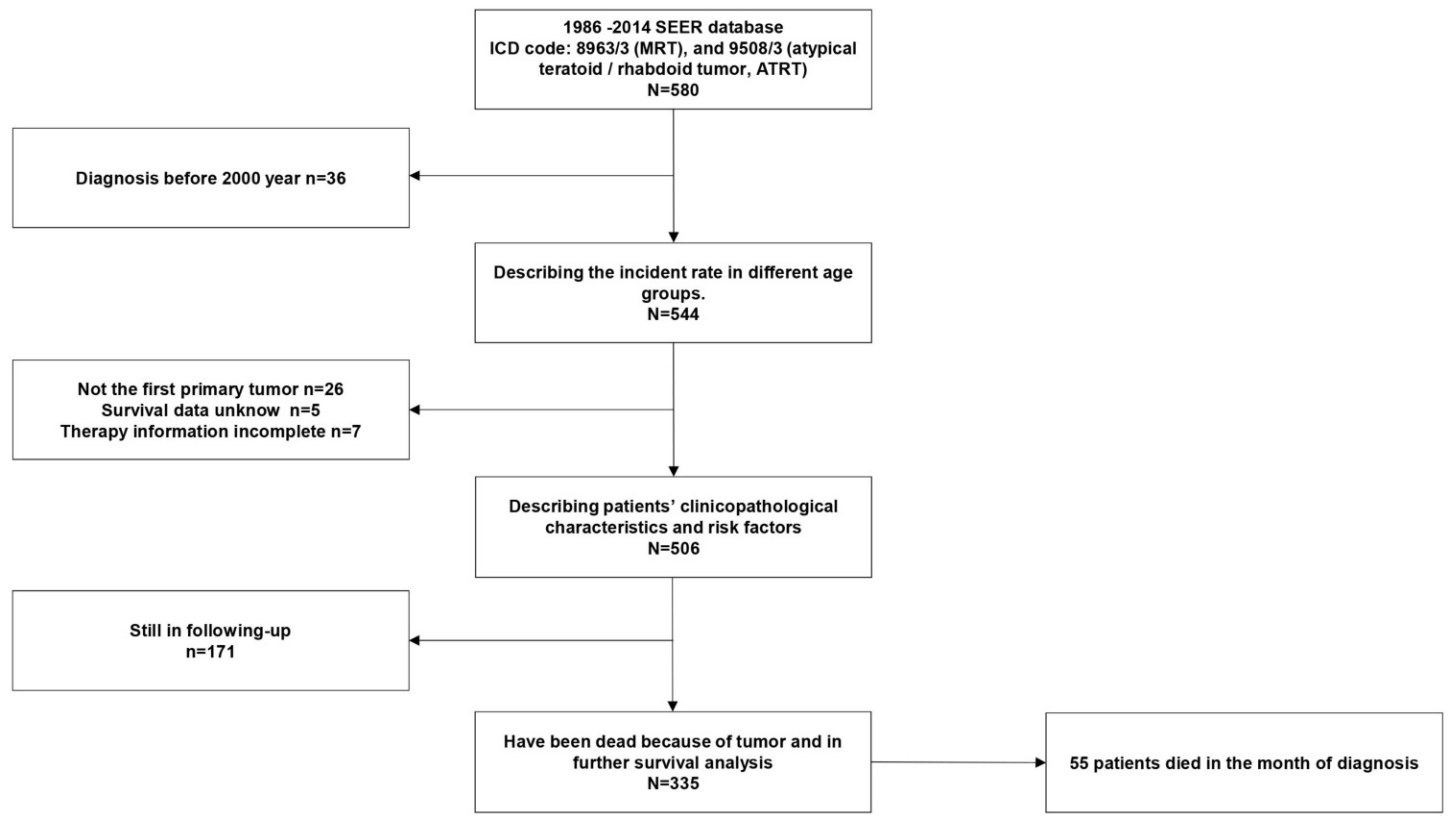
Flow chart of selecting patients.

**Figure 2:**
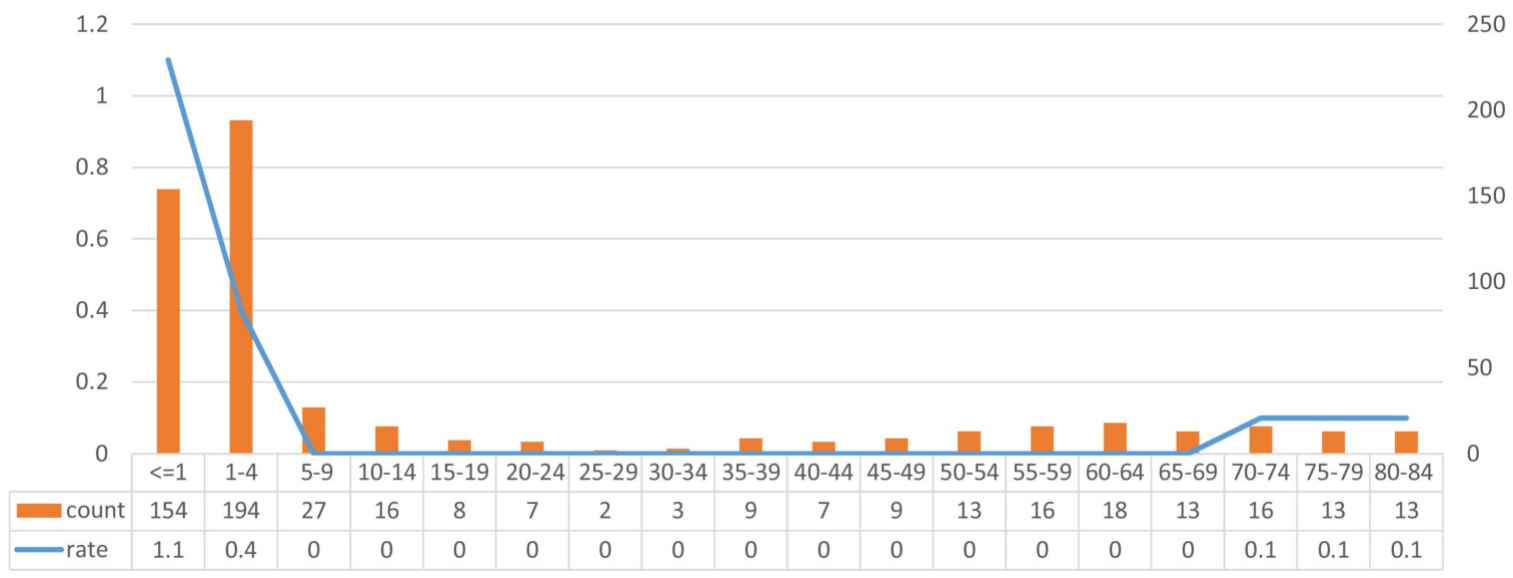
Incident of different age groups.

**Figure 3:**
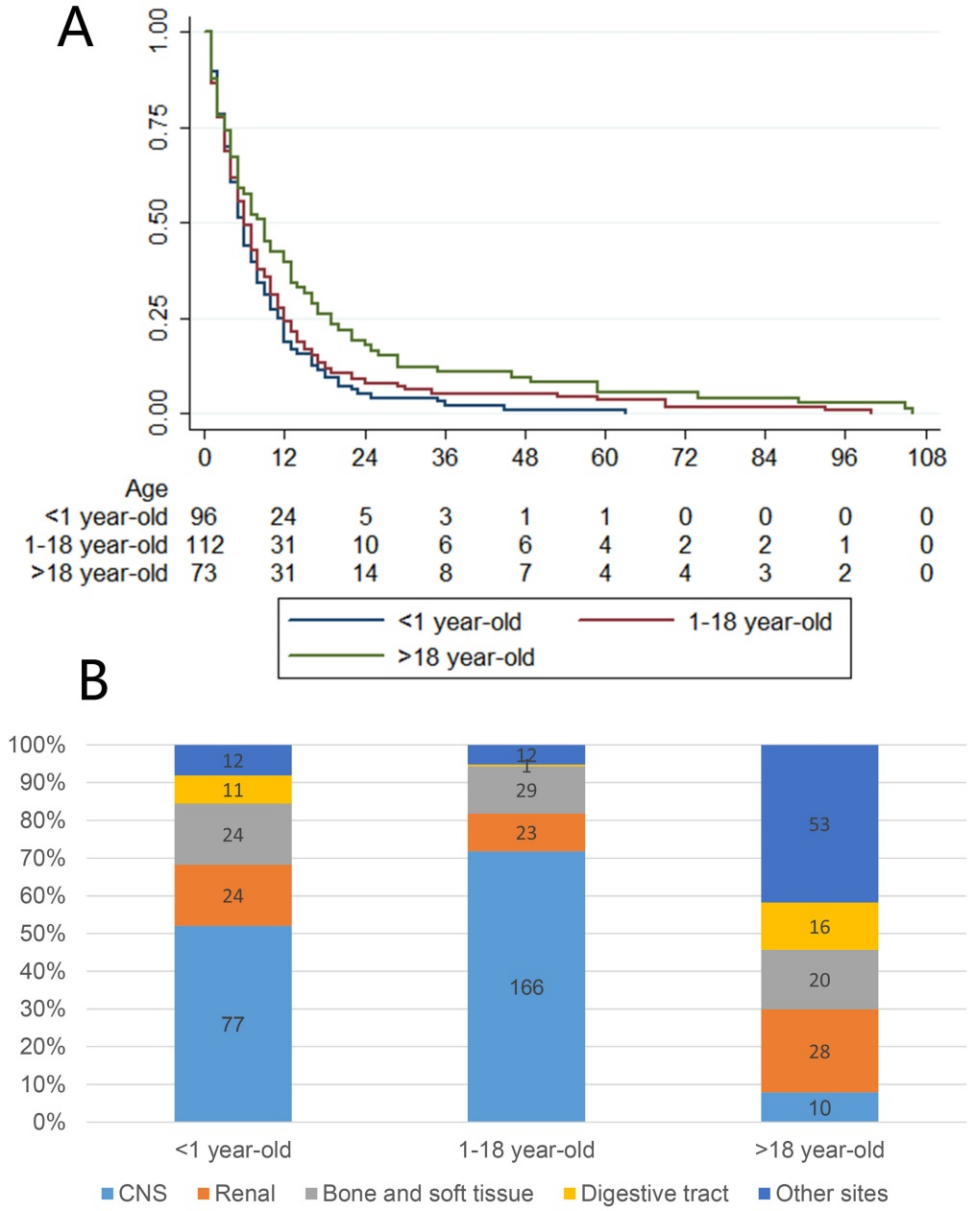
A: Survival of different age groups. B: Different primary site composition of different age groups.

**Figure 4:**
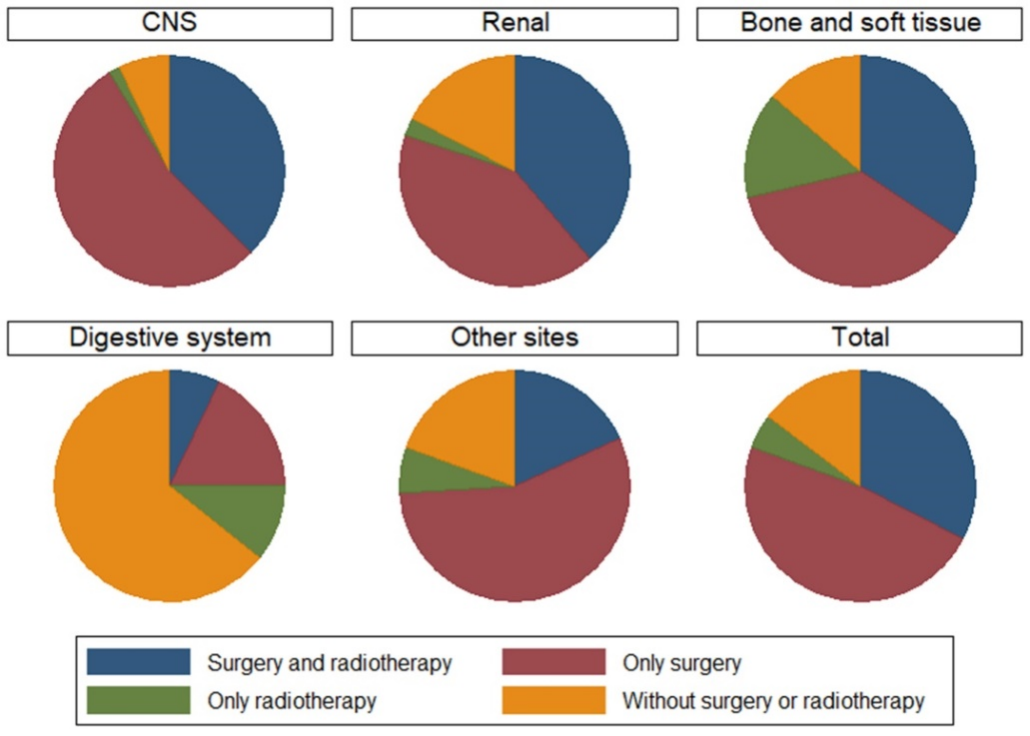
The treatment choices of different primary sites.

**Figure 5:**
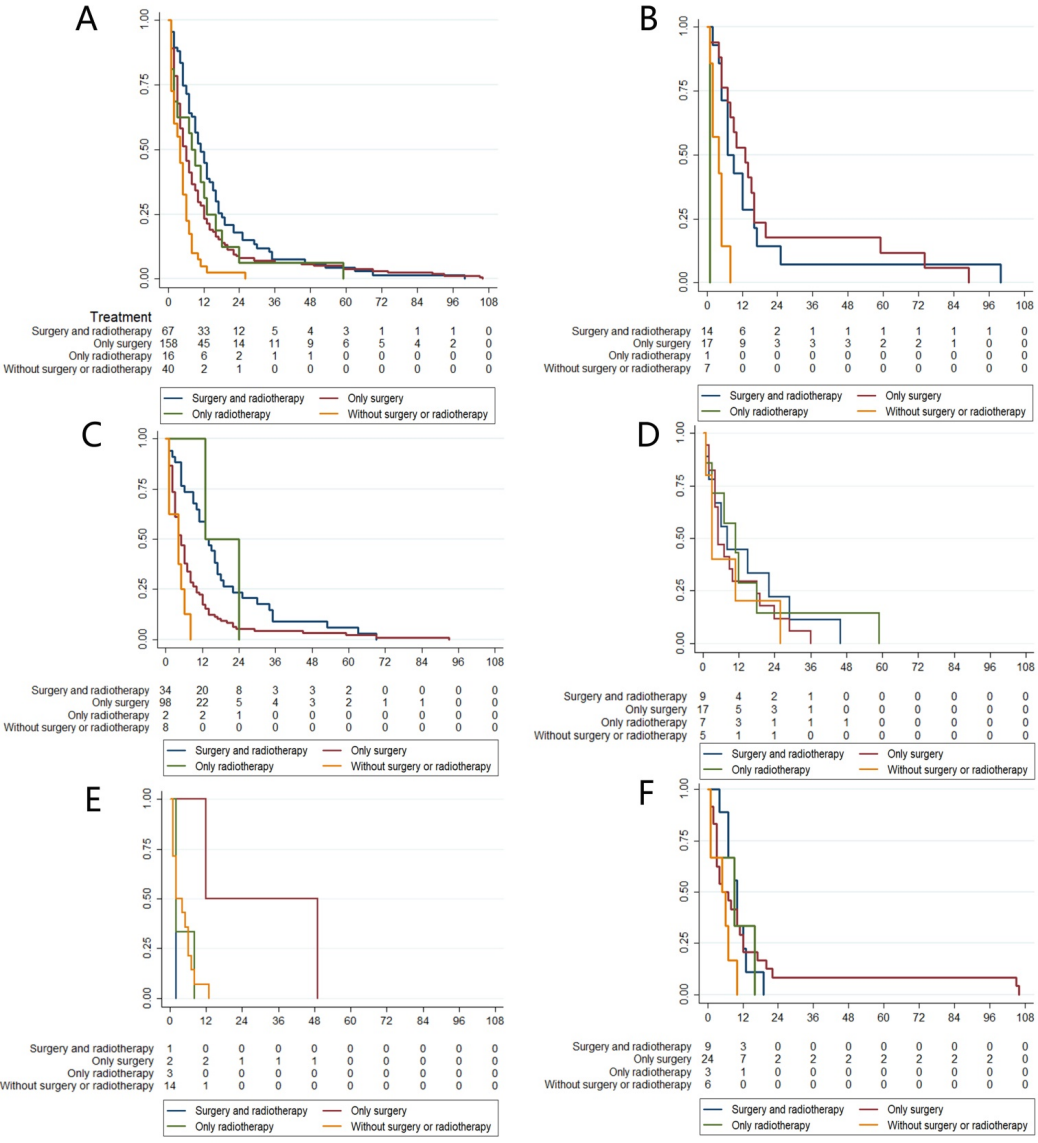
The relationship between treatment choices and overall survival in different primary sites. A: Treatment choices and different survival of all MRTs. B: Treatment choices and different survival of renal MRTs. C: Treatment choices and different survival of CNS MRTs. D: Treatment choices and different survival of bone and soft tissue MRTs. E: Treatment choices and different survival of digestive tract MRTs. F: Treatment choices and different survival of other uncommon sites MRTs.

The SEER database of the National Cancer Institute (NCI) is a source of information on cancer incidence and survival. The SEER database is publicly available for studies of cancer-based epidemiology and health policy (http://seer.cancer.gov/). We retrieved data from the SEER database based on the November 2017 submission of patients diagnosed with MRTs. We used the SEER*Stat 8.3.5 program to identify the frequency of in different age groups and individuals with a reported diagnosis of MRT in the SEER database based on the ICD-O-3 with the following morphological classifications: 8963/3 (MRT) and 9508/3 (ATRT) 23. There was no limitation with regard to the primary site. We excluded patients who met the following criteria: 1) patients diagnosed before 2000 because of the unified definition of MRT; 2) patients whose survival data were incomplete; 3) patients in whom MRT was not the primary tumor; 4) patients whose treatment information was incomplete; and 5) patients who were still in follow-up for the purpose of the survival analysis. We included only patients who developed MRTs for survival and regression analyses. A flow diagram of the selection process is presented in Fig 1. A description of the SEER staging system is as follows: localized stage (confined entirely to the organ of origin), regional stage (extending beyond the organ of origin and/or regional nodal spread), and distant stage (distant metastasis or extension). Patients who did not fit or could not be staged by this system were recorded as “unknown”. 24 The primary sites in the digestive system included the liver, esophagus, stomach, pancreas, intestine, colon and rectum.

### Statistical analysis

Mean values are used to describe continuous data, with discrete variables displayed as totals and frequencies. The patients' demographic data and tumor characteristics are summarized with descriptive statistics. Comparisons of categorical variables among the different groups of patients were performed using the chi-square test. Death attributed to MRT was considered the endpoint. Survival function estimates and comparisons among different variables were performed using the Kaplan-Meier method and the log-rank test. A Cox proportional hazards model was used to compare the effects of prognostic variables on survival. All statistical analyses were performed using Intercooled Stata 12.0 (Stata Corporation, College Station, TX, USA). Statistical significance was considered for results with a two-sided *P* value<0.05.

## Figures and Tables

**Table 1:** Patient basic characteristics

	Renal (%)	CNS (%)	Bone and Soft Tissue (%)		Digestive System (%)	Other Sites (%)	Total Number	*P-*value
**Mean survival month**	13.80 (7.23-20.37)	9.43 (7.28-11.58)		10.26 (6.48-14.04)	5.58 (1.58-9.58)	9.65 (4.34-14.96)		
**Mean age, year-old**	27.24 (17.76-36.71)	2.80 (1.62-3.96)		19.35 (10.89-27.81)	34.83 (20.78-48.88)	45.09 (36.70-53.48)		
**Age group**								<0.01
< 1 year-old	24 (32.00)	77 (30.43)		24 (32.88)	11 (39.29)	12 (15.58)	148	
1-18 year-old	23 (30.67)	166 (65.61)		29 (39.73)	1 (3.57)	12 (15.58)	231	
>18 year-old	28 (37.33)	10 (3.95)		20 (27.40)	16 (57.14)	53 (68.83)	127	
**Gender**								0.60
Male	31 (41.33)	109 (43.08)		31 (42.47)	15 (53.57)	39 (50.65)	225	
Female	44 (58.67)	144 (56.92)		42 (57.53)	13 (46.43)	38 (49.35)	281	
**Race**								0.35
Black	60 (80.00)	196 (77.47)		51 (69.86)	24 (85.71)	61 (79.22)	392	
White	12 (16.00)	29 (11.46)		12 (16.44)	1 (3.57)	10 (12.99)	64	
Others	3 (4.00)	28 (11.07)		10 (13.00)	3 (10.71)	6 (7.79)	50	
**Marrial status**								<0.01
Married	19 (25.33)	3 (1.19)		14 (19.18)	9 (32.14)	28 (36.36)	73	
Unmarried	52 (69.33)	249 (98.42)		55 (75.34)	15 (53.57)	35 (45.45)	406	
Other	3 (4.00)	1 (0.40)		3 (4.11)	4 (14.29)	11 (14.29)	22	
Unspecific	1 (1.33)	0 (0.00)		1 (1.37)	0 (0.00)	3 (3.90)	5	
**SEER stage**								<0.01
Localized	1 (1.33)	21 (8.30)		3 (4.11)	3 (10.71)	1 (1.30)	29	
Regional	8 (10.67)	6 (2.37)		8 (10.96)	1 (3.57)	2 (2.60)	25	
Distant	7 (9.33)	10 (3.95)		9 (12.33)	4 (14.29)	13 (16.88)	43	
Unknow	59 (78.67)	216 (85.38)		53 (72.60)	20 (71.43)	61 (79.22)	409	
							
**Following-up status**								0.14
Activated	87 (34.39)	30 (40.00)		27 (36.99)	4 (14.29)	23 (29.87)	171	
Finished	166 (65.61)	45 (60.00)		46 (63.01)	24 (86.71)	54 (70.13)	335	
**Primary site surgery**								<0.01
Yes	60 (80.00)	231 (91.30)		52 (71.23)	7 (25.00)	57 (74.03)	407	
No	15 (20.00)	22 (8.70)		21 (28.77)	21 (75.00)	20 (25.97)	99	
**Radiotherapy**								<0.01
Yes	44 (58.67)	154 (60.87)		37 (50.68)	23 (82.14)	58 (75.32)	316	
No	31 (41.33)	99 (39.13)		36 (49.32)	5 (17.86)	19 (24.68)	190	
								

**Table 2:** Univariate analyses on different primary sites identifying risk factors

	Renal		CNS		Bone and Soft Tissue		Digestive System		Other Sites	
	HR (95%CI)	*P-*value	HR (95%CI)	*P-*value	HR (95%CI)	*P-*value	HR (95%CI)	*P-*value	HR (95%CI)	*P-*value
**Gender**	0.72 (0.38-1.38)	0.32	0.83 (0.59-1.15)	0.26	0.64 (0.33-1.21)	0.17	1.32 (0.54-3.23)	0.54	1.57 (0.85-2.88)	0.15
**Race**	0.99 (0.81-1.24)	1.00	1.03 (0.97-1.10)	0.36	0.99 (0.88-1.11)	0.84	1.07 (0.91-1.25)	0.42	0.90 (0.70-1.14)	0.37
**Marrial status**	0.99 (0.77-1.30)	0.99	1.16 (0.37-3.70)	0.80	0.88 (0.49-1.56)	0.65	0.94 (0.53-1.69)	0.84	1.25 (0.91-1.72)	0.18
**SEER Stage**	1.07 (0.95-1.20)	0.30	1.03 (0.96-1.09)	0.43	0.92 (0.84-1.01)	0.08	1.12 (0.96-1.31)	0.14	1.02 (0.92-1.14)	0.69
**Age group**										
< 1 year old	Reference		Reference		Reference		Reference		Reference	
1-18 year old	0.39 (0.16-0.95)	0.04	0.55 (0.39-0.78)	<0.01	0.35 (0.16-0.74)	0.01	1.10 (0.14-8.96)	0.93	0.42 (0.15-1.20)	0.11
> 18 year old	1.01 (0.49-2.07)	0.99	0.57 (0.25-1.34)	0.20	0.51 (0.23-1.14)	0.10	0.74 (0.29-1.89)	0.53	0.69 (0.32-1.49)	0.35
**Primary site surgery**										
No	Reference		Reference		Reference		Reference		Reference	
Yes	0.10 (0.04-0.25)	<0.01	0.60 (0.32-1.15)	0.13	0.60 (0.30-1.19)	0.15	0.10 (0.02-0.48)	<0.01	0.50 (0.24-1.07)	0.08
**Radiotherapy**										
No	Reference		Reference		Reference		Reference		Reference	
Yes	0.73 (0.38-1.39)	0.34	0.23 (0.16-0.34)	<0.01	0.45 (0.23-0.86)	0.02	1.02 (0.34-3.11)	0.96	0.75 (0.38-1.46)	0.39

**Table 3:** Multivariate analyses on different primary sites identifying risk factors

	Renal		CNS		Bone and Soft Tissue		Digestive System		Other Sites	
	HR (95%CI)	*P-*value	HR (95%CI)	*P-*value	HR (95%CI)	*P-*value	HR (95%CI)	*P*-value	HR (95%CI)	*P*-value
**Gender**	0.87 (0.44-1.74)	0.70	0.79 (0.56-1.12)	0.19	0.80 (0.40-1.61)	0.53	1.97 (0.59-6.58)	0.27	1.80 (0.94-3.44)	0.08
**Race**	0.93 (0.73-1.12)	0.54	0.99 (0.93-1.06)	0.84	1.03 (0.90-1.18)	0.65	1.02 (0.82-1.26)	0.89	0.92 (0.71-1.20)	0.55
**Marrial status**	1.02 (0.80-1.30)	0.88	1.74 (0.51-5.96)	0.38	0.78 (0.34-1.82)	0.57	0.26 (0.08-0.85)	0.03	1.23 (0.93-1.63)	0.15
**SEER Stage**	1.13 (0.98-1.30)	0.10	1.02 (0.96-1.09)	0.55	0.87 (0.78-0.97)	0.01	1.01 (0.82-1.25)	0.90	1.02 (0.91-1.15)	0.73
**Age group**										
< 1 year old	Reference		Reference		Reference		Reference		Reference	
1-18 year old	0.23 (0.09-0.58)	<0.01	0.77 (0.53-1.11)	0.16	0.34 (0.14-0.84)	0.02	0.52 (0.05-5.68)	0.59	0.53 (0.17-1.67)	0.28
> 18 year old	0.96 (0.42-2.20)	0.93	1.41 (0.55-3.65)	0.47	0.49 (0.17-1.38)	0.18	0.57 (0.15-2.14)	0.40	0.74(0.32-1.68)	0.47
**Primary site surgery**										
No	Reference		Reference		Reference		Reference		Reference	
Yes	0.06 (0.02-0.17)	<0.01	0.60 (0.31-1.18)	0.14	0.50 (0.24-1.07)	0.07	0.03 (0.01-0.28)	<0.01	0.58 (0.24-1.40)	0.23
**Radiotherapy**										
No	Reference		Reference		Reference		Reference		Reference	
Yes	2.34 (1.01-5.43)	0.05	0.22 (0.15-0.34)	<0.01	0.44 (0.21-0.90)	0.03	3.78 (0.65-22.10)	0.14	0.67 (0.31-1.47)	0.32

## References

[1] Haas JE, Palmer NF, Weinberg AG, Beckwith JB (1981). Ultrastructure of malignant rhabdoid tumor of the kidney. A distinctive renal tumor of children. HUM PATHOL.

[2] Gonzalez-Crussi F, Goldschmidt RA, Hsueh W, Trujillo YP (1982). Infantile sarcoma with intracytoplasmic filamentous inclusions: distinctive tumor of possible histiocytic origin. CANCER-AM CANCER SOC.

[3] Tekautz TM, Fuller CE, Blaney S, Fouladi M, Broniscer A, Merchant TE (2005). Atypical teratoid/rhabdoid tumors (ATRT): improved survival in children 3 years of age and older with radiation therapy and high-dose alkylator-based chemotherapy. J CLIN ONCOL.

[4] Maschek H, Werner M, Busche G, Weinel P (1992). [Congenital rhabdoid tumor in the mediastinum and liver. Case report and review of the literature]. PATHOLOGE.

[5] Hilden JM, Meerbaum S, Burger P, Finlay J, Janss A, Scheithauer BW (2004). Central Nervous System Atypical Teratoid/Rhabdoid Tumor: Results of Therapy in Children Enrolled in a Registry. J CLIN ONCOL.

[6] Woehrer A, Slavc I, Waldhoer T, Heinzl H, Zielonke N, Czech T (2010). Incidence of atypical teratoid/rhabdoid tumors in children: a population-based study by the Austrian Brain Tumor Registry, 1996-2006. CANCER-AM CANCER SOC.

[7] Harris NL, Jaffe ES, Diebold J, Flandrin G, Muller-Hermelink HK, Vardiman J (2000). The World Health Organization classification of neoplasms of the hematopoietic and lymphoid tissues: report of the Clinical Advisory Committee meeting-Airlie House, Virginia, November, 1997. Hematol J.

[8] Kemi M (2018). D, Alfred R, Julie A S. Practical Guide to Surgical Data Sets: Surveillance, Epidemiology, and End Results (SEER) Database. JAMA SURG.

[9] Sultan I, Qaddoumi I, Rodríguez-Galindo C, Nassan AA, Ghandour K, Al-Hussaini M (2010). Age, stage, and radiotherapy, but not primary tumor site, affects the outcome of patients with malignant rhabdoid tumors. PEDIATR BLOOD CANCER.

[10] Pai Panandiker AS, Merchant TE, Beltran C, Wu S, Sharma S, Boop FA (2012). Sequencing of Local Therapy Affects the Pattern of Treatment Failure and Survival in Children With Atypical Teratoid Rhabdoid Tumors of the Central Nervous System. International Journal of Radiation Oncology Biology Physics.

[11] Tomlinson GE, Breslow NE, Dome J, Guthrie KA, Norkool P, Li S (2005). Rhabdoid Tumor of the Kidney in The National Wilms' Tumor Study: Age at Diagnosis As a Prognostic Factor. J CLIN ONCOL.

[12] Morozov A, Lee SJ, Zhang ZK, Cimica V, Zagzag D, Kalpana GV (2007). INI1 induces interferon signaling and spindle checkpoint in rhabdoid tumors. CLIN CANCER RES.

[13] Venneti S, Le P, Martinez D, Eaton KW, Shyam N, Jordan-Sciutto KL (2011). p16INK4A and p14ARF tumor suppressor pathways are deregulated in malignant rhabdoid tumors. J Neuropathol Exp Neurol.

[14] Martina A F, Yura G, Simon B, Daniel W (2020). Translational genomics of malignant rhabdoid tumours: Current impact and future possibilities. SEMIN CANCER BIOL.

[15] Puri DR, Meyers PA, Kraus DH, Laquaglia MP, Wexler LH, Wolden SL (2008). Radiotherapy in the multimodal treatment of extrarenal extracranial malignant rhabdoid tumors. PEDIATR BLOOD CANCER.

[16] Squire SE, Chan MD, Marcus KJ (2007). Atypical teratoid/rhabdoid tumor: the controversy behind radiation therapy. J Neurooncol.

[17] Ginn KF, Gajjar A (2012). Atypical teratoid rhabdoid tumor: current therapy and future directions. FRONT ONCOL.

[18] Lafay-Cousin L, Hawkins C, Carret AS, Johnston D, Zelcer S, Wilson B (2012). Central nervous system atypical teratoid rhabdoid tumours: the Canadian Paediatric Brain Tumour Consortium experience. EUR J CANCER.

[19] Chen Y, Wong T, Ho DM, Huang P, Chang K, Shiau C (2006). Impact of radiotherapy for pediatric CNS atypical teratoid/rhabdoid tumor (single institute experience). International Journal of Radiation Oncology Biology Physics.

[20] Kenichi K, Yoshinao O (2017). Oncogenic roles of SMARCB1/INI1 and its deficient tumors. CANCER SCI.

[21] Saunders J, Ingley K, Wang XQ, Harvey M, Armstrong L, Ng T (2020). Loss of BRG1 (SMARCA4) Immunoexpression in a Pediatric Non-Central Nervous System Tumor Cohort. PEDIATR DEVEL PATHOL.

[22] von Hoff K, Hinkes B, Dannenmann-Stern E, von Bueren AO, Warmuth-Metz M, Soerensen N (2011). Frequency, Risk-Factors and Survival of Children With Atypical Teratoid Rhabdoid Tumors (AT/RT) of the CNS Diagnosed between 1988 and 2004, and Registered to the German HIT Database. PEDIATR BLOOD CANCER.

[23] Surveillance Research Program, National Cancer Institute SEERStat software (.

[24] Adamo M, Dickie L, Ruhl J (2016). SEER Program Coding and Staging Manual 2016. National Cancer Institute, Bethesda, MD 20850-9765.

